# Liver Abscesses Caused by Streptococcus intermedius in an Immunocompromised Patient

**DOI:** 10.7759/cureus.2107

**Published:** 2018-01-24

**Authors:** Sireesha Reddy, Kalbir Singh, Susan Hughes

**Affiliations:** 1 University of California, San Francisco – Fresno Department of Family & Community Medicine; 2 Internal Medicine/ Residency Program, San Joaquin General Hospital; 3 Hospice and Palliative Medicine, University of California, San Francisco – Fresno Department of Family & Community Medicine

**Keywords:** streptococcus intermedius, immunocompromised

## Abstract

Streptococcus intermedius is a Gram-positive bacterium that is part of the normal flora in the oral cavity, as well as in the upper respiratory, female urogenital, and gastrointestinal tracts. It may also be found in human feces and is the dominant species found in subgingival plaque. We present a case of a 50-year-old man who came to the emergency room with abdominal pain for weeks associated with nausea and vomiting, diarrhea, and fever. A computed tomography (CT) scan of the abdomen showed numerous cysts throughout the liver. The patient was started on intravenous (IV) antibiotics and fluid from two of the liver abscesses was obtained for culture.

## Introduction

Streptococcus intermedius is a Gram-positive bacterium that is part of the normal flora in the oral cavity, as well as in the upper respiratory, female urogenital, and gastrointestinal tracts. It may also be found in human feces and is the dominant species found in subgingival plaque.

The “Streptococcus millerigroup” (SMG) has been known by a variety of names and, in the past, has been considered a single species that is loosely synonymous with Streptococcus (S.) anginosus. Members of the SMG are now separated into three distinct species—Streptococcus intermedius, Streptococcus constellatus, and Streptococcus anginosus—with S. constellatus and S. intermedius being more closely related to each other than to S. anginosus. However, because many phenotypic tests for the characterization of these species yield similar results, the identification of isolates can be difficult. S. intermedius tends to be associated with an infection of the head, neck, and respiratory tract. One of the most striking features of species in the SMG is their tendency to cause abscesses. However, both the potential of each of the SMG species to cause abscesses and the clinical features of the cases associated with abscesses have not been addressed in depth with regard to well-identified strains [1].

Streptococcus intermedius is usually found as a solitary isolate associated with deep-seated purulent abscesses, typically found in the brain or liver, central nervous system infections, and infective endocarditis [2-6]. Other articles concluded that undiagnosed diabetes, undiagnosed squamous cell carcinoma, and parenteral drug abusers were common among patients who tested positive for Streptococcus intermedius [2-8].

## Case presentation

**Figure 1:**
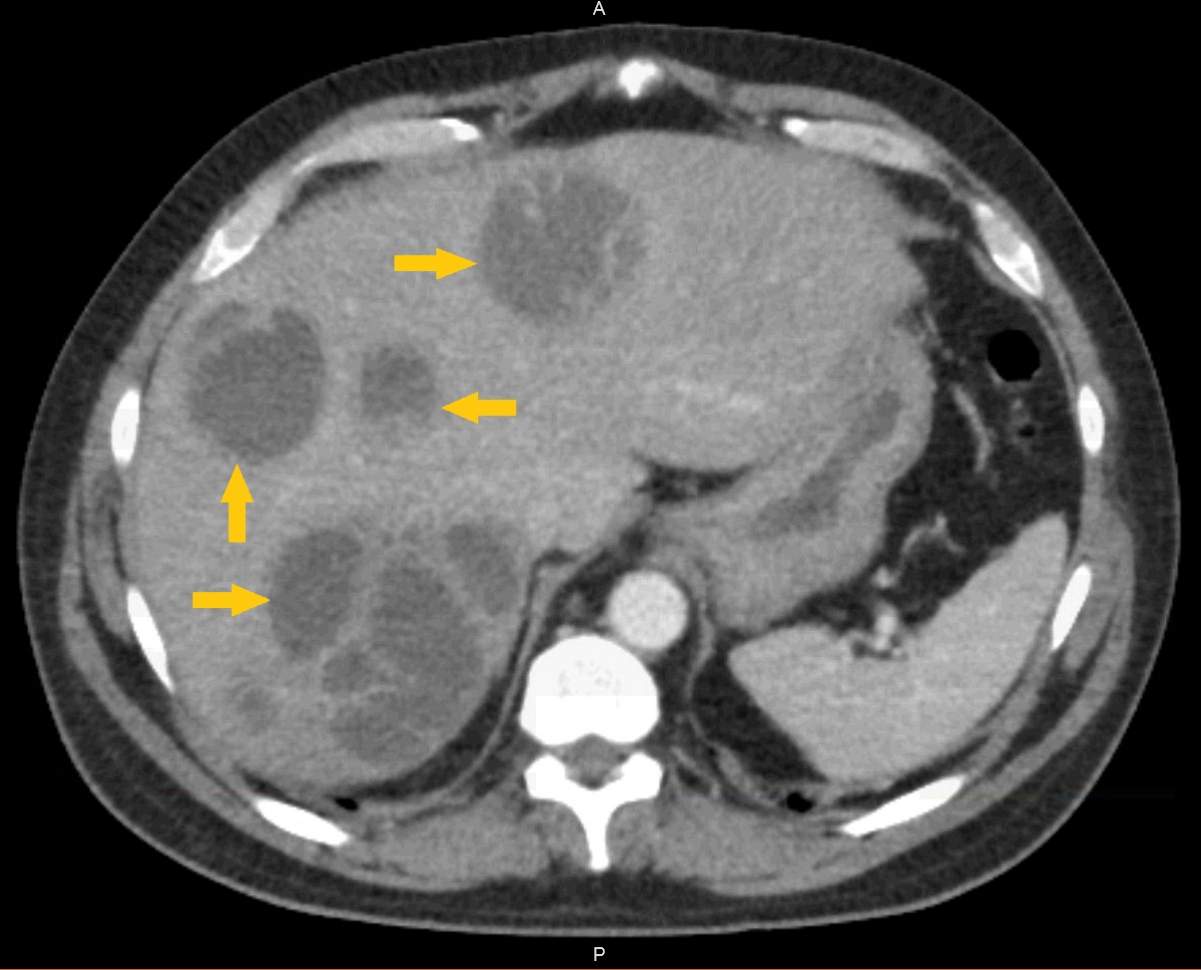
Initial CT scan of abdomen with arrows indicating multiple liver abscesses CT: computed tomography

A 50-year-old man came to the emergency room with generalized abdominal pain for weeks associated with nausea, vomiting, diarrhea, and fever. Abnormal lab findings included white blood cells (WBCs): 21.0 (4-11 per microliter), total bilirubin: 2.9 (0.3-1.2 mg/dl), aspartate aminotransferase (AST): 62 (8-40 U/L), alanine transaminase (ALT): 75 (10-40 U/L), and alkaline phosphatase: 168 (25-100 U/L). The emergency room provider ordered a CT scan of the abdomen. Findings for this patient, as shown in Figure 1, were “numerous, complex, predominantly cystic masses seen throughout the liver; 4 cm cystic collection interposed between the tip of the right lobe of the liver and hepatic flexure of the colon.” At this point, the patient was admitted and symptomatic, and empiric treatments of IV levofloxacin and IV metronidazole were started.

Further history of this patient: no travel, no pet exposure, but two teeth extracted one month prior to admission. A diagnosis of Streptococcus intermedius was made from the drainage and culture of the fluid from the liver abscesses, which the lab noted with a failure to thrive for antibiotic susceptibility testing. The blood culture grew Entamoeba histolytica, but it was not detected in the stool.

**Figure 2:**
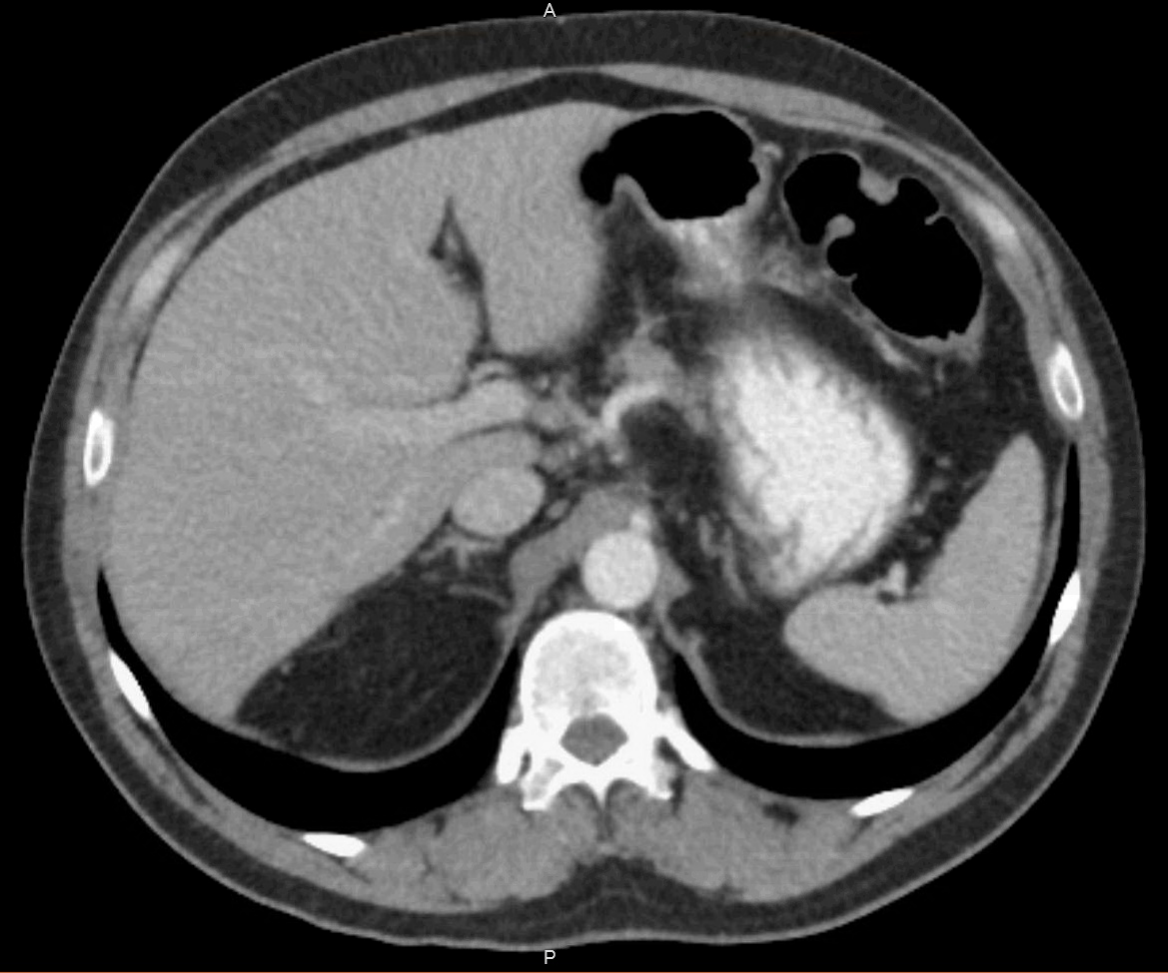
CT scan of abdomen after nine months CT: computed tomography

Our patient was treated initially with IV levofloxacin and metronidazole for two weeks, which was then changed to IV meropenem per the recommendation of the infectious disease specialist. Further abscesses were drained by an interventional radiologist at the University of California, San Francisco. The patient was afebrile after starting IV meropenem. Meropenem was given for a total of six weeks. A CT scan of the abdomen approximately nine months after initial admission showed complete resolution of the hepatic abscesses, as shown in Figure 2.

## Discussion

The unusual findings for this case led us to conduct a further study on other cases of Streptococcus intermedius in our area. We obtained a list of 18 patients testing positive for Streptococcus intermedius from the Community Regional Medical Center, Fresno, California, and Adventist Medical Center, Hanford, California, laboratories from 2010-2015.

We identified patients by a retrospective electronic medical record review. Data collected included verification for Streptococcus intermedius from respective cyst/abscess drainage, abscess location, immune status, co-morbidities, and history of exposures including, but not limited to, dental work, food, or travel.

We defined immunocompromised as any patient with coronary artery disease, diabetes mellitus, cancer, intravenous drug use, human immunodeficiency virus/acquired immunodeficiency syndrome (HIV/AIDS), or hypertension [9].

The risk factors leading to the immunocompromised state of our sample and the development of abscesses were the following:

·         56% had chronic conditions (diabetes mellitus type 2, hypertension, AIDS, or cancer).

·         33% had lifestyle choices (alcohol use, tobacco use, raw beef intake, or intravenous drug abuse).

·         11% had a surgical intervention (dental work or laparoscopic cholecystectomy).

The majority of cases were detected in 2015 (n=7).

Previous studies have shown this uncommon organism involves mainly diabetic patients and parenteral drug abusers with an intra-abdominal suppurative source in the skin and soft tissues. It is commonly isolated from patients with endocarditis, peritonitis, emphysema, and hepatic and appendiceal abscesses. An aggressive course of antibiotics and control of co-morbidities (e.g., diabetes and hypertension) might lead to earlier symptom-free status in patients with dental issues [2-8].

There were several limitations to our study. First, it was retrospective and the documentation of risk factors was done by multiple providers. The small number of cases limited the possibility of defining a relationship between abscess locations and risk factors. An increase in cases in 2015 was likely due to the ability to do full speciation of Streptococcal anginosus. Finally, the duration of risk factors varied as well as the length and state of chronic conditions in individual patients.

## Conclusions

The direct relationship between Streptococcus intermedius as a bacterium with the development of abscesses is not always clear. However, this case report demonstrates a possible association of positive Streptococcus intermedius abscesses developing in patients who are in some form of immunocompromised state.

To understand the etiology of this bacteria, further studies are needed to correlate a relationship between immune status and bacteria.
